# Why We Share: A Systematic Review of Knowledge-Sharing Intentions on Social Media

**DOI:** 10.3390/bs14080636

**Published:** 2024-07-25

**Authors:** Jia Hu, Shuhaida Md Noor

**Affiliations:** School of Communication, Universiti Sains Malaysia, Gelugor 11800, Malaysia; imhujia@student.usm.my

**Keywords:** social media, knowledge-sharing intention, systematic review, ROSES

## Abstract

Social media’s potential for knowledge dissemination is under-utilized due to limited user participation. This study systematically reviews factors affecting knowledge-sharing intentions on social platforms using the ROSES protocol. We searched Scopus and Web of Science for quality, relevance, and rigor, finding that 65% of the articles shared were published in high-quality journals (Q1 or Q2), with the Journal of Knowledge Management accounting for 15%. Since 2015, 62.5% of research has been published, highlighting increased activity. Quantitative methods dominated (95%), with Zhihu being the most studied platform. We identified four key themes—psychological, technological, environmental, and social—covering 47 determinants centered on trust and attitude, primarily based on individual and social behavior theories. This is the first systematic exploration of elements influencing knowledge-sharing intentions on social media, providing insights to enhance user interaction and guide social media strategies in knowledge-centric organizations.

## 1. Introduction

Knowledge is regarded as the essential element of competition between and survival and development of organizations and countries [1]. It differs from other forms of information (such as numbers, symbols, artwork, or activities) because it results from gaining insight and experience, and applying information meaningfully [2]. Oranga divides personal knowledge into tacit knowledge and explicit knowledge [3]. Tacit knowledge, which is not inherently quantifiable, is commonly defined as “know-how” stored exclusively in individuals’ minds, such as oral skills, aesthetic sense, or innovation [4]. Explicit knowledge is structured knowledge that can be easily recorded, stored, described, and transmitted electronically, such as regulations, procedures, technical papers, articles, manuals, formulations, and patents [5]. Knowledge acquisition and utilization serve as critical strategic assets for individuals and organizations, enabling them to establish and sustain a competitive edge [6]. Knowledge sharing is essential to expanding the spread of knowledge and extending the benefits of that knowledge. Connecting individuals is an efficient technique to encourage and facilitate knowledge sharing [7,8].

### 1.1. Social Media as a Tool for Knowledge Sharing

The advent of social media has profoundly impacted interpersonal communication, information dissemination, and collaborative efforts [9]. It is considered a vital knowledge-sharing tool because it brings together many users who can easily exchange knowledge and express ideas, experiences, and perceptions [10]. Moreover, enterprises can utilize social media platforms to generate and distribute their content, thereby capitalizing on employee and customer-pooled knowledge and insights [11]. Social media have not only driven innovation and efficiency in the fields of healthcare [12,13] and education [14,15], but also play an essential role in various other domains, such as tourism [16,17] and business [18,19]. The widespread application of social media accelerates the dissemination of knowledge, diversifies access channels, and promotes global knowledge sharing and innovation. The practical implications of social media platforms in knowledge sharing cannot be underestimated. However, people are often surprisingly reluctant to share knowledge on social media [20,21,22]. Therefore, factors influencing the intention to use social media as knowledge-sharing platforms warrant investigation.

In their widely referenced work, Kaplan and Heinlein define social media as “… a group of Internet-based applications that build on the ideological and technological foundations of Web 2.0, and that allow the creation and exchange of User Generated Content” [10] (p. 61). Aichner et al. note that the term exchange has since 2010 been increasingly replaced with the term share [23]. We argue that while exchange denotes expecting something back in return, share indicates motivation to offer something to others without necessarily expecting reciprocity and is thus more aligned with the knowledge-sharing definition used in this study. Notably, the semantic technologies in the emerging Web 3.0 offer a better experience for individuals and machines to connect, share, and use knowledge innovatively [24], promising a more vibrant and conducive environment for knowledge sharing. Additionally, based on emerging technologies such as artificial intelligence, data science, and blockchain, social media demonstrate immense potential in enhancing knowledge sharing. Artificial intelligence improves the efficiency and accuracy of knowledge acquisition through personalized recommendations, automated content generation, and intelligent search systems [25]. Data science leverages big data analysis and user behavior analytics to optimize content distribution strategies and ensures the authenticity of shared knowledge through sentiment analysis [26]. Meanwhile, blockchain technology offers a decentralized and secure method for storing and sharing knowledge, protects intellectual property, and verifies the credibility of information [27]. Overall, integrating these technologies significantly enhances the effectiveness of knowledge sharing on social media platforms, promoting the global dissemination and innovation of knowledge.

### 1.2. Challenges in Knowledge Sharing on Social Media

Following Shayne [28], the social media considered in this study are those that include social networks (e.g., Facebook and Twitter), messaging apps (e.g., WhatsApp and WeChat), photo/media sharing (e.g., Instagram and YouTube), blogging and publishing networks (e.g., Weibo and HubSpot), interactive apps (e.g., Snapchat and TikTok), discussion forums (e.g., Quora and Reddit), bookmarking and content curation (e.g., Pinterest and Flipboard), review networks (e.g., Yelp and TripAdvisor), social shopping networks (e.g., Etsy and Faveable), interest-based networks (e.g., Goodreads and Houzz), sharing economy networks (e.g., Uber and Airbnb), audio-only apps (e.g., Clubhouse and Spotify), and anonymous social networks (e.g., Whisper and 4chan). Based on the data provided by Statista, global social media users reached 4.95 billion in 2023 [29]. In 2022, the average daily social media usage worldwide was 151 min daily [30]. Furthermore, social media users are expected to increase to almost 6 billion in 2027 [30]. Social media attracts users mainly because people use it to keep in touch with friends and family, to fill their free time, to read news stories, to share their knowledge, or to discuss their opinions with others [31].

Social media has changed how people communicate, share content, interact, and collaborate, and is an essential source for seeking knowledge in various fields [32]. According to Meltwater [31], 30.3% of adults use social media to find relevant information. For example, students use social media to gain professional knowledge [33], patients use social media to find health advice [34], tourists use social media to plan their travel routes [35], and consumers use social media to find information about products [36]. Social media, therefore, has become an invaluable platform for knowledge sharing at both individual and organizational levels [37]. For this reason, it is significant to encourage people to become more engaged in knowledge sharing on social media [38].

Nevertheless, sharing knowledge on social media remains challenging for some people [39]. Vuori and Okkonen have identified three obstacles to knowledge sharing: (1) not knowing that the knowledge they have is valuable to others, (2) not having the motivation to share it, and (3) not having a suitable medium through which to share that knowledge [40]. Consequently, several studies have been undertaken to understand knowledge-sharing intentions on social media (KSIoSM) [9,41,42]. These studies assume that intentions are a prerequisite for action [43]. The Theory of Planned Behavior (TPB) posits that the association between intention and action is supported by the influence of attitudes, subjective norms, and behavioral control on these intents [43]. Therefore, it is vital to understand the factors that increase the intention to share knowledge via social media.

Despite the increasing use of social media for knowledge seeking and sharing, there is a significant gap in our understanding of the factors that influence knowledge-sharing intentions on these platforms. Previous research has primarily focused on individual behaviors, and few have taken into account other influences, such as social, technological, and environmental contexts. Identifying all of the contributing factors is critical to consolidating existing knowledge and guiding future research. This systematic review aims to fill this gap by synthesizing extant literature and providing a clear direction for enhancing knowledge-sharing practices on social media.

### 1.3. Current Psychology

Numerous studies have reported many factors influencing knowledge sharing through social media [9,42,44,45,46]. Nevertheless, few studies have attempted to sort or categorize the influencing factors identified in the KSIoSM literature. Systematic literature reviews can help to provide researchers with a comprehensive understanding of a research topic that has already been extensively explored and can elucidate gaps in the research warranting further investigation. This study is the first to use a systematic literature review to examine the factors influencing KSIoSM. With this at its core, the authors also make a comprehensive summary of KSIoSM research, including the publication year, journal publication, research methods, research theories, and social media platforms, mainly addressing the following questions (RQs):

**RQ1.**  *Which social media platforms are commonly examined in studies on KSIoSM?*

**RQ2.**  *Which theoretical frameworks are utilized to analyze KSIoSM?*

**RQ3.**  *What factors influence individuals’ KSIoSM?*

The remainder of this review article is structured as follows. The next section discusses the underlying principles behind the methods used in this review assessment. Following this, we present the study’s results in Section 3, construct a conceptual model of the influencing factors in Section 4, and discuss the classification of these factors in Section 5. Finally, we provide a summary of the results in Section 6.

## 2. Methodology

### 2.1. Review Protocol

Haddaway et al. proposed the Reporting Standards for Synthesizing Systematic Evidence (ROSES) to enhance and manage the overall quality of systematic literature reviews [47]. We have chosen the ROSES approach as a guide, considering the high transparency apparent throughout the review process. These standards originated from environmental management literature and were designed to accommodate the complexities and variations found in various contexts and studies related to synthesis methods [47]. ROSES has been used in fields as diverse as disaster management [48], tourism management [49], and knowledge sharing [50]. As such, we believe that ROSES may be equally appropriate to the present investigation. Per ROSES, the systematic review process consists of four steps. In the first step, we applied the PICo method to define the research question, where the “P” refers to the Problem or Population, the “I” stands for Interest, and the “Co” is for Context. In the second step, we performed a three-stage literature search. These three stages entailed identification, screening, and ascertaining eligibility. The third step in the study involved evaluating the quality of the literature. We used the Mixed-Method Appraisal Tool (MMAT) version 2018 developed by McGill University, Canada as proposed by Hong et al. [51], to facilitate our quality evaluation. Articles assessed to have sufficient quality were deemed appropriate for inclusion in the review. Following data extraction from the final sample of articles, we used thematic synthesis to analyze the extracted data.

### 2.2. Formulation of the Study Question

We used the PICo tool to establish the research questions for this study. The PICo tool is intended to facilitate the development of research questions appropriate for systematic reviews by focusing on the Problem or Population, Interest, and Context [52]. Using PICo, our research question identifies individuals (Population), intentions to share knowledge on social media (Interest), and influencing factors (Context) as part of the synthesis, thus: “What are the factors that influence individuals’ intention to share knowledge on social media”?

### 2.3. Question Search Strategy

**Figure 1:**
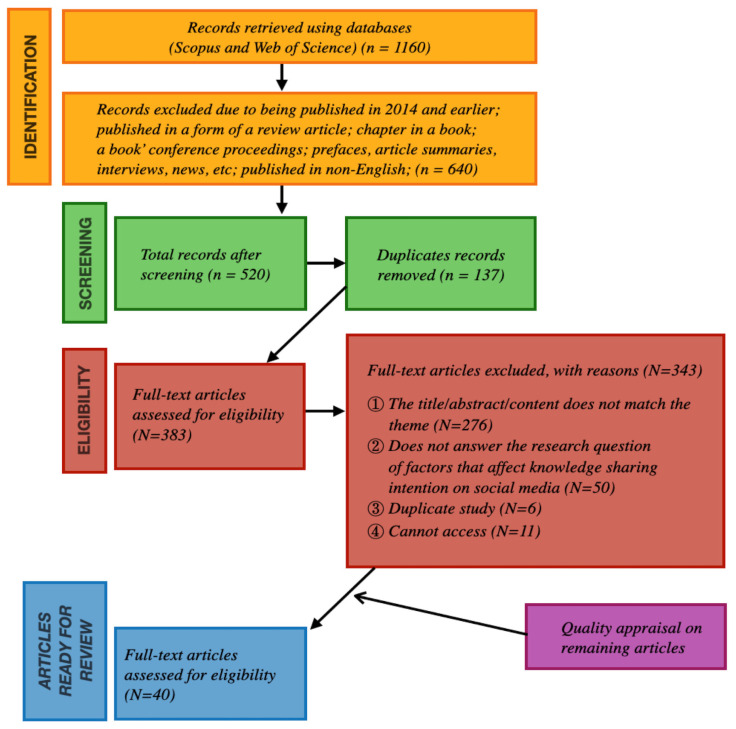
Systematic review process flowchart.

Following protocols outlined by Shaffril et al. [53], we sourced publications for this study via a three-step process of identification, screening, and determining eligibility (see Figure 1). This process enables researchers to effectively identify and integrate relevant studies, thus producing systematic reviews that are thorough, well-structured, and transparent. A further explanation of this procedure is provided in Appendix A.

### 2.4. Quality Appraisal

For this systematic review, the researchers combined data from various studies with the support of MMAT, a widely used and proven hybrid methodological systematic evaluation tool [51]. MMAT facilitates the integration of data from five types of studies: quantitative descriptive research, qualitative research, randomized controlled trials, non-randomized research, and mixed methods research [51]. We evaluated the selected studies’ quality, reliability, and validity according to the MMAT criteria. These criteria encompass evaluating the research question’s ability to yield sufficient data, aligning the data collection methods with the research questions, data sources, data collection procedures, analysis techniques, and interpretation of findings. After analysis, all of the 40 articles selected for this systematic review met the MMAT criteria. For details, see Appendix B.

### 2.5. Data Extraction and Analyses

Thematic analysis was conducted in light of the review’s reliance on varying research designs, aiming to identify the most effective approaches for integrating the divergent findings through qualitative synthesis [54]. This study used the qualitative synthesis technique described by Flemming et al. [55], which emphasizes the suitability of the thematic synthesis approach for integrating data from different study methodologies, implemented following the methodological standards proposed by Kiger and Varpio [56]. Thematic analysis aims to identify and elucidate recurring patterns in previous studies by identifying similarities or connections in the provided data [57]. Initially, the researchers engaged in a comprehensive examination of the entire dataset through repeated readings. This procedure gave the researchers significant insights into the unprocessed data and established the basis for all subsequent actions.

The subsequent step involved the generation of preliminary codes. After thoroughly reviewing the selected articles, the researchers extracted any relevant data related to the original research question. The inductive coding framework was used to systematically reveal document patterns, commonalities, and associations in the collected data to tease out relevant themes, which need to have a strong correlation with the core data and embrace the entirety of the dataset [57]. Throughout this procedure, a total of four primary themes were produced. Subsequently, the researchers iteratively applied the process to ascertain potential sub-themes for each article, culminating in identifying 30 distinct sub-themes encompassing 47 influencing factors. These themes and sub-themes were then verified by two experts in the field of knowledge sharing to determine their relevance to the research study, resulting in the identification of four main themes and a total of 30 sub-themes.

## 3. Results

### 3.1. Journal Landscape

**Figure 2:**
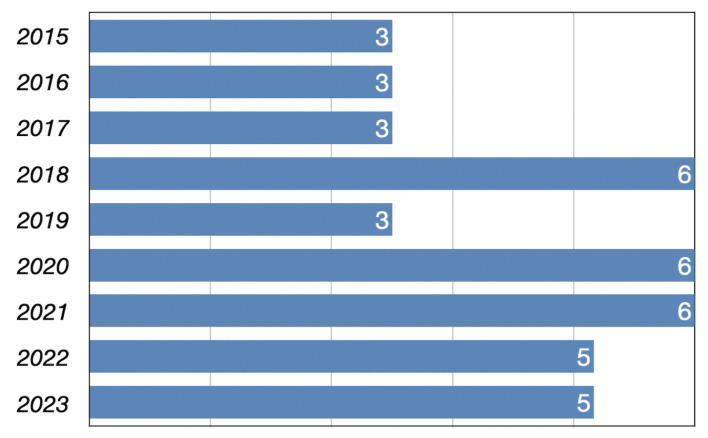
Publication years of selected articles. The numbers to the right of this chart represent the number of relevant papers published in that year.

Forty papers on factors influencing KSIoSM were scrutinized in the present study. The selected journals and their rankings are shown in Table 1. Most selected articles (65%; n = 26) were drawn from high-quality journals in both Scopus and Web of Science databases, ranked Q1 or Q2. The *Journal of Knowledge Management* has been found to possess the highest volume of articles on the topic of KSIoSM, accounting for six articles or 15% of the overall corpus [58,59,60,61,62,63]. The following five journals are *Aslib Journal of Information Management* [64,65], *Behavior and Information Technology* [66,67], *International Journal of Human-Computer* [41,46], *Information Systems Management* [68,69], and *Sustainability* [70,71], all involving two articles and 5%. The remaining 24 journals have published at least one paper about KSIoSM [9,23,36,44,45,63,72,73,74,75,76,77,78,79,80,81,82,83,84,85,86,87,88,89,90], all with a percentage of 2.5%. The survey also showed that 62.5% (n = 25) of the 40 articles on the topic since 2015 were published in the last five years (see Figure 2). This discovery furnishes valuable insights into contemporary trends in the research landscape about KSIoSM. High-caliber scholarly journals assert dominance within this domain, emphasizing the *Journal of Knowledge Management*. Additionally, there exists a conspicuous upward trajectory in research endeavors within this sphere in recent years, underscoring an escalating interest in and scholarly engagement with KSIoSM.

### 3.2. Research Method

**Figure 3:**
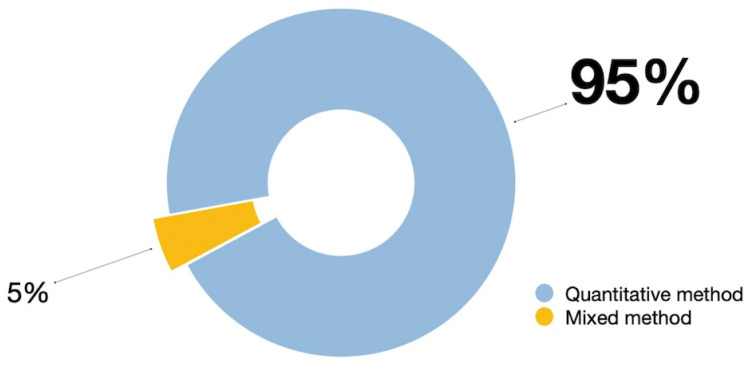
Research methods of selected journals.

Notably, 95% of the articles (n = 38) used quantitative methods, including thirty-six articles using survey-based questionnaires [9,23,36,41,44,45,46,58,60,61,62,63,64,65,66,67,68,70,71,72,73,74,75,76,77,78,79,80,81,82,83,84,85,86,87,88,89,90], one article using self-administered questionnaires [69], and one article using structured questionnaire surveys [59]. The remaining two articles take a hybrid approach, with one using interviews, text mining, and survey-based questionnaires [71] and the other using survey-based questionnaires and in-depth interviews [89] (see Figure 3). This result indicates the popularity of quantitative methods in examining the willingness to share knowledge on social media, highlighting the significant dependence on statistical and numerical analysis in this field.

### 3.3. Usage of Social Media

**Figure 4:**
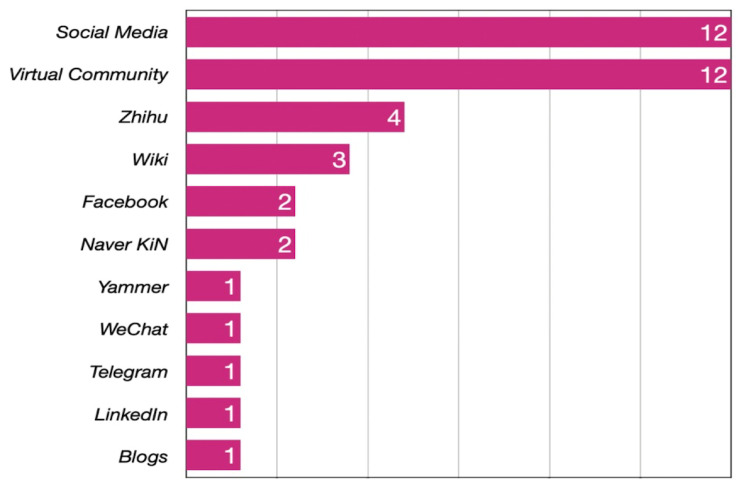
Study count by social media type. The numbers on the right of this chart represent the number of studies using this type of platform.

Figure 4 shows the types of social media discussed in the studies identified in this review. It indicates that social media and virtual communities are the categories of research that have received more attention from scholars, with 12 relevant publications each. In addition, as the popularity and importance of social media have increased, studies have become increasingly focused on specific social media tools. Zhihu is the most studied social media platform [23,41,67,86], followed by Wiki [58,78,84], Facebook [66,76], and Naver KiN [64,65]. Other social media tools include Yammer [88], WeChat [85], Telegram [82], LinkedIn [71], and Blogs [69]. When aggregated, it was found that 70% of the studies in this review (n = 28) focused on social media, indicating a growing awareness that social media comprises a viable knowledge-sharing tool in academia.

### 3.4. Theories Lens

As shown in Table 2, of the 40 articles, 19 theories were used, mainly focusing on two categories: personal behavior theories and social behavior theories. The former aims to explain KSIoSM on a personal/individual level. Table 2 shows that this group contains 11 theories, among which the Theory of Planned Behavior (TPB) [9,61,65,66,72], Social Cognitive Theory (SCT) [41,67,75,77], Intrinsic and Extrinsic Motives (IET) [46,61,74,77], Technology Acceptance Model (TAM) [9,45,69,70,72], Unified Theory of Acceptance and Use of Technology (UTAUT) [63,81,83,84], and Theory of Reasoned Action (TRA) [59,65,90] are the most concerned. These theories highlight key psychological factors such as attitudes, self-efficacy, and trust, which significantly influence individuals’ intentions to share knowledge on social media [9,59,61,66,68,70,72,73,74,78,82,83,84,85,86,88,90].

Table 2 shows that previous studies adopted seven theories to explain individuals’ KSIoSM in a social context. Social Exchange Theory (SET) [59,62,74,87] is the most adopted theory. Social behavior theories emphasize the importance of social factors, such as social identity, social capital, and social influence, in shaping knowledge-sharing intentions [23,46,63,75,76,84].

It is worth noting that some studies combined these two types of theories to more fully explain the determinants of KSIoSM, such as blending SET and Commitment Trust theory [62], TPB and Social Identity Theory [66], TRA and SET [59], IET and SET [74], TPB and TAM [72], and Information Systems Continuous Use Model and Commitment Trust Theory [73]. These combinations allow for a more holistic understanding of how psychological and social factors interact to influence KSIoSM.

However, ten articles did not describe any theory [36,44,64,68,71,78,79,80,82,85]. The scholarly focus is predominantly on the theory of personal behavior and is relatively less focused on the social dimension, in particular, the combination of the two theoretical types is rare. Worse, the theories of mass communication used to explain people’s specific behavior toward social media, such as the Use and Gratification Theory and Para-Social Interaction, are not mentioned. This gap suggests an under-utilization of mass communication theories, which could provide valuable insights into how media-specific factors influence knowledge-sharing behavior on social media platforms.

## 4. Conceptual Model

Based on the literature review and the survey findings in Table 3, the factors influencing KSIoSM can be categorized into four main groups.

The first category pertains to psychological factors, which encompass attitudes [9,59,61,72,74,84,90], trust [68,70,82,85], self-efficacy [66,83,86,88], satisfaction [64,65], commitment [62,68,73,87], perceived behavioral control [9,61,72], knowledge-related factors [41,45,46,58,60,86,90], and various motivational aspects [69,71,77,82]. These psychological factors are crucial because they directly affect an individual’s cognitive processes and attitudes towards knowledge sharing. For instance, trust and self-efficacy enhance the confidence and willingness to share knowledge [70,82,83], while attitudes and perceived behavioral control influence the intention and perceived ease of sharing [59,61]. Notably, the primary reason for categorizing knowledge-related factors as psychological is that elements such as the importance of knowledge exchange, level of knowledge, source credibility, content credibility, knowledge codification effort, and advertisement content likeability are linked to individual cognitive processes and attitudes. Since psychological factors encompass motivation, perception, learning, and attitude or belief system [91], it is reasonable to classify them as psychological.

The second category comprises technological factors, which include performance expectancy [63,81,83,84], perceived usefulness [60,69], perceived ease of use [36,89], task–technology fit [88], virtual network connectivity [79], face risk [46], and experience using social media platforms [60]. Technological factors are vital as they determine the usability and effectiveness of the platforms used for knowledge sharing. Performance expectancy and perceived usefulness influence how beneficial users perceive the technology to be [60,81], while perceived ease of use and task–technology fit ensure that users find the platform convenient and suitable for their needs [88,89]. These factors relate to the various technologies and technical contexts in which knowledge is created, stored, transmitted, and shared [92].

The third category addresses environmental factors, which refers to the external environment and conditions related to knowledge sharing, encompassing group norms [76], community climate [67], community’s need for knowledge [58], community identification [78], community attachment [80], managerial support [69], perceived organizational support [71], leader–member exchange [71], fairness [41], and institutional factors [71]. Environmental factors create a supportive or hindering backdrop for knowledge sharing. For example, a positive community climate and strong managerial support can foster a culture of sharing [67,69], while fairness and institutional factors can ensure equitable and trustworthy exchanges [41,71].

Lastly, subjective norms [9,61,76,90], social influence [46,63,84], social identity [23,46,76], and social capital [23,75] can be summarized as social factors that influence the social context and interpersonal relationship of knowledge sharing [93]. Social factors are significant as they shape the social dynamics and relational aspects that encourage or inhibit sharing. Social influence and subjective norms pressure individuals to conform to group behaviors [9,46,61,63,76,84,90], while social identity and social capital build networks and relationships that facilitate knowledge exchange [23,46,75,76].

**Figure 5:**
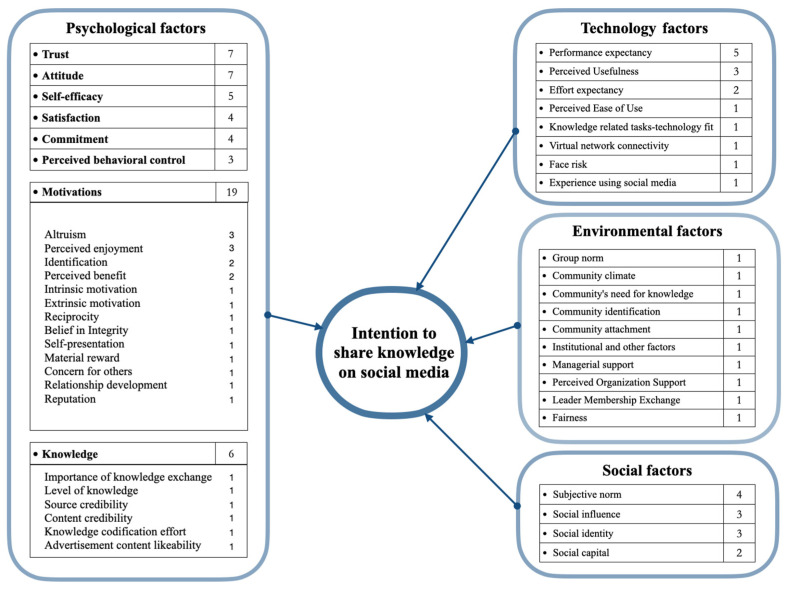
Conceptual framework. In the antecedent section, the numbers reflect the papers supporting those antecedents’ influence on KSIoSM. The above factors positively impact the intentions to share knowledge on social media.

Drawing from this review above, Figure 5 presents the study’s conceptual framework, illustrating the interrelationships among these factors influencing KSIoSM.

## 5. Discussion

This paper aims to conduct a comprehensive literature review focusing on the determinants influencing KSIoSM. The review delineates four primary categories of factors crucial for understanding knowledge-sharing success: psychological, technological, environmental, and social.

### 5.1. Psychological Factors

Table 4 summarizes the literature investigating the psychological factors influencing KSIoSM. Of the 40 studies, 25 psychological factors were identified (see Figure 5 and Table 4). Seven papers separately reported that trust and attitude influence KSIoSM, making them the most frequently cited factors. Trust was identified as the most effective component in motivating people to share knowledge online [37]. Hoseini, Saghafi, and Aghayi reported that trust positively affects the propensity to share knowledge via mobile social networks [82]. Similarly, Ahmed and Khurshid expanded on the C-TAM-TPB model to include numerous factors, including the interpersonal trust factor, noting that interpersonal trust positively influences behavioral willingness to use social media to share knowledge about disaster relief [9]. Moreover, as a crucial component of TPB, TRA, and TAM, attitudes contribute to explaining knowledge-sharing intention, as numerous studies have demonstrated [9,83,90]. In particular, research on knowledge sharing in the social media setting emphasizes the potential importance of attitudes in predicting the intention to share knowledge utilizing social media [9,59,61,72,74,84,90]. These studies observe that having a favorable attitude about sharing knowledge via social media leads to a positive intention to share knowledge. Aside from trust, attitude is among the most frequently mentioned factors.

Other influential factors, according to the studies in this review, include self-efficacy [66,68,83,86,88], satisfaction [64,65,73,88], commitment [62,68,73,87], and perceived behavioral control [9,61,72]. Furthermore, other factors related to motivations include altruism [69,82,90], perceived enjoyment [41,46,81], identification [41,66], perceived benefit [46,58], intrinsic motivation [77], extrinsic motivation [77], reciprocity [82], belief in integrity [36], self-presentation [46], material reward [86], concern for others [71], relationship development [46], and reputation [82]. Other studies have reported that knowledge itself is an influential factor that cannot be ignored; this includes the level of knowledge [58], the importance of knowledge exchange [60], knowledge codification efforts [58], source credibility [85], content credibility [85], and advertisement content likeability [45].

On social media, psychological elements like trust and attitude are ubiquitous and critical, directing the willingness to share knowledge. Trust enhances the inclination to engage, and a positive attitude often translates into a higher propensity to share [9]. Given the complexity and interconnectivity of these factors, trust and attitude are especially critical areas for further research. Their significant impact on KSIoSM suggests that platforms and researchers should focus more on strategies to enhance trust and foster positive attitudes towards knowledge sharing. The thorough investigation of these and other factors, such as self-efficacy and satisfaction, highlights their interconnected roles in creating an environment favorable to exchanging knowledge. This cohesive body of research offers a detailed understanding of how individual psychology shapes the online knowledge-sharing landscape. By addressing these psychological components, platforms can better align with user needs and expectations, ultimately encouraging more frequent and meaningful knowledge sharing. This understanding helps in developing strategies that not only attract users but also sustain their engagement over time, ensuring a vibrant and collaborative online community.

### 5.2. Technology Factors

Based on the technology acceptance model, eight factors were classified as technological factors, including performance expectancy [63,67,81,83,84], perceived usefulness [60,69,89], effort expectancy [63,84], perceived ease of use [36,89], knowledge-related task–technology fit [88], virtual network connectivity [79], face risk [46], and experience using social media [60], as shown in Table 5.

The review results showed that performance expectancy is an essential antecedent to people’s KSIoSM, and five studies found that performance expectancy is positively correlated to KSIoSM [63,67,81,83,84]. In addition, face risk is considered a disadvantage of sharing knowledge on social media and can inhibit people’s willingness to share [46]. However, from a technological standpoint, numerous studies support the impact of perceived usefulness, effort expectancy, perceived ease of use, knowledge-related task–technology fit, virtual network connectivity, face risk, and social media usage experience on people’s KSIoSM [36,46,60,63,79,88].

Overall, seamlessly integrating technology with user goals and user-centric design is critical to encouraging active knowledge sharing on social media platforms. Technological factors facilitate knowledge-sharing intentions and reveal the importance of social media platform design and functional optimization. Users’ perceived usefulness and ease of use directly affect their willingness to share knowledge on these platforms, while technical adaptation of knowledge-related tasks and virtual network connectivity further enhance the effectiveness of platforms.

Among these factors, performance expectancy and perceived usefulness stand out as particularly worthy of further study due to their significant influence on users’ willingness to share knowledge. Performance expectancy refers to the degree to which an individual believes that using a particular system will help them to achieve gains in job performance, which is crucial in determining the perceived benefits of knowledge sharing on social media [81,83]. Perceived usefulness, which is the degree to which a person believes that using a particular system would enhance their job performance, is another critical factor that significantly impacts users’ motivation to engage in knowledge-sharing activities [60,69].

Additionally, the role of virtual network connectivity in enhancing knowledge sharing is a crucial area for further research. Virtual network connectivity provides the infrastructure for seamless information exchange and collaboration, making it a fundamental component of social media platforms that support knowledge sharing [79]. Understanding how to optimize virtual network connectivity can help platforms to design more efficient and effective systems for facilitating knowledge exchange.

Together, these technical factors analyzed by the technology acceptance model provide valuable insights into how to promote knowledge sharing through technological means. Optimizing these factors can help social media platforms to design more user-friendly and robust systems that motivate users to participate actively in knowledge sharing.

### 5.3. Environmental Factors

Environmental factors that impact the dissemination of knowledge are frequently concentrated within organizations, including organizational culture and environment, support from management, and organizational structures [67]. Table 6 summarizes the literature investigating environmental factors affecting KSIoSM. Ten of the forty studies comprising this review identified ten environmental factors.

Li, Tang, and Chau found that perceived organization support, leader membership exchange, and institutional factors benefit people’s willingness to contribute knowledge about building safety across the Internet of Things [71]. Similarly, Cai and Shi investigated the mechanism by which community climate influences KSIoSM, finding that fairness, recognition, and openness influence the willingness of users to share their knowledge [67]. Cai, Yang, and Shi emphasized the critical role of fairness in users’ online contribution behavior, finding that the willingness to share knowledge in online communities was higher among those who were fond of sharing and perceived greater overall levels of fairness [41]. Moreover, several studies reported that group norms, the community’s need for knowledge, community identification, community attachment, and managerial support are critical environmental factors influencing the willingness to share knowledge on social media [58,69,76,78,80].

Among these factors, community identification and managerial support stand out as particularly worthy of further study. Community identification refers to the degree to which individuals feel a sense of belonging and identity within a community, which can significantly impact their willingness to share knowledge [78]. Research has shown that stronger community identification leads to higher engagement in knowledge-sharing activities [78,80]. Managerial support, which includes encouragement and resources provided by organizational leaders, is also a critical factor that influences employees’ and community members’ knowledge-sharing intentions [69]. Studies have demonstrated that supportive management practices can enhance individuals’ motivation to share knowledge by creating a positive and conducive environment for knowledge exchange [69,71].

These findings suggest that nurturing and fair environments are equally essential to incentivizing knowledge sharing on social media platforms, underscoring the need for organizations to foster a supportive culture that values and encourages open knowledge exchange.

Overall, environmental factors are crucial in shaping users’ knowledge-sharing intentions. An open, fair, and supportive organizational culture can significantly increase the willingness of employees and community members to share knowledge. Therefore, organizations should aim to establish and maintain an environment conducive to knowledge sharing to facilitate broader and more effective knowledge dissemination.

### 5.4. Social Factors

Individuals are influenced by various social factors, such as subjective norms, social capital, and peer influence, rooted in interpersonal interactions and broader societal structures and processes [94]. Several studies have found that social factors are essential prerequisites for people’s willingness to share knowledge on social media, including subjective norms, social identity, social capital, and social influence [9,23,46,61,63,75,76,84,90].

Subjective norms pertain to the perceived social influence that individuals experience, which can either encourage or discourage engagement in a particular action [43]. It denotes an individual’s subjective interpretations of specific behavioral patterns that hold significance for them [71]. In their study, Liao employed social influence theory to investigate the antecedent model of knowledge-sharing intention among virtual communities [76]. The results show that subjective norms profoundly influence behavioral choice [76]. Arif, Qaisar, and Kanwal [90], Ahmed and Khurshid [9], and Nguyen, Malik, and Sharma [61] conducted similar studies.

Furthermore, social influence [46,63,84], social identity [23,46,76], and social capital [23,75] are significant social factors that affect an individual’s KSIoSM, as shown in Table 7. Among these, social capital and social identity stand out as particularly worthy of further study. Social capital refers to the resources available to individuals through their social networks, including trust, norms, and networks that can improve the efficiency of society by facilitating coordinated actions [75]. Research has shown that higher levels of social capital lead to greater knowledge-sharing intentions due to the trust and reciprocal relationships built within social networks [23,75]. Social identity, which involves individuals’ identification with a particular group and the extent to which they see themselves as part of that group, also significantly impacts their willingness to share knowledge [76]. Studies have indicated that a stronger social identity within online communities leads to higher engagement and more active knowledge-sharing behaviors [23,46,76].

These social dynamics highlight the importance of fostering robust, positive social interactions on platforms to create an environment that welcomes and actively seeks knowledge sharing. By encouraging healthy social relationships and community identity, social media platforms can effectively increase users’ willingness to share knowledge, promoting widespread dissemination and utilization of knowledge.

## 6. Conclusions

This study offers a groundbreaking and systematic analysis of the factors influencing individuals’ intentions to share knowledge on social media, addressing significant gaps in the existing literature. Utilizing the ROSES protocol and comprehensively reviewing sources from Scopus and Web of Science, this research provides a holistic perspective that synthesizes findings across various domains, contrasting with the fragmented nature of previous studies. The analysis confirms the importance of psychological factors, such as trust and attitudes, aligning with established behavioral theories like TPB and SCT. Technological determinants, often focused on usability and functionality in prior research, are systematically categorized here, highlighting their impact on user participation. Environmental and social factors are also more thoroughly examined, emphasizing the need for supportive environments and fostering cultures of knowledge sharing. Notably, the study reveals that behavioral theories, rather than mass communication frameworks, predominantly influence knowledge-sharing intentions, suggesting a shift in focus for future research and applications. The findings are highly relevant, offering actionable insights for enhancing user interaction, encouraging knowledge sharing, and guiding social media strategies for knowledge-centric organizations. This study fills literature, methodological, and knowledge gaps by providing a consolidated view and a detailed framework of 47 determinants, serving as a foundation for future research and extending the understanding of knowledge dissemination on social media.

This study represents the inaugural systematic assessment of factors influencing individuals’ KSIoSM. The insights gleaned from our analysis hold significant implications for users seeking to enhance their social interactions and organizations endeavoring to leverage social media as platforms for knowledge dissemination. By elucidating the multifaceted determinants of knowledge-sharing behavior, our findings offer valuable guidance for social media managers who foster knowledge-centric organizational environments. In summation, this study advances scholarly discourse on knowledge-sharing in the digital age and underscores the importance of continued research efforts to elucidate the intricate interplay between individual motivations, social dynamics, and technological affordances in shaping online knowledge-sharing behaviors. We anticipate that our findings will catalyze future investigations to enhance our understanding of knowledge-sharing phenomena within social media, thereby facilitating the development of more effective strategies for fostering collaborative knowledge exchange in online environments.

While our systematic review provides valuable insights into knowledge sharing on social media, it is essential to recognize its limitations. Firstly, our analysis relies on existing literature, which may have publication bias and methodological constraints. Moreover, we only considered articles from Scopus and Web of Science, potentially excluding relevant research from other sources. Additionally, our findings may not be widely applicable due to the western-centric focus of the literature. These limitations highlight the need for future research to address gaps in our understanding. For instance, scholars could integrate mass communication theories to better understand knowledge-sharing behaviors. Longitudinal studies could track changes in these behaviors over time, revealing nuanced patterns. Cross-cultural comparisons could shed light on cultural influences, while investigating emerging technologies like virtual and augmented reality platforms could offer insights into their impact on knowledge dissemination. Furthermore, research on organizational interventions to promote a knowledge-sharing culture is crucial. Given the above shortcomings, future researchers can use different methods, data sources, and new research perspectives to explore this field further. Addressing these areas will deepen our understanding of knowledge-sharing dynamics on social media and inform the development of practical strategies for collaborative knowledge exchange in digital environments.

## Figures and Tables

**Table 1:** Selected journals and their rankings.

Journal	Total Number of Selected Articles (%)	Indexed by WoS	WoS Quartile	Indexed by Scopus	Scopus Quartile
*Journal of Knowledge Management*	6 (15%)	√	Q1	√	Q1
*Aslib Journal of Information Management*	2 (5%)	√	Q3	√	Q2
*Behavior and Information Technology*	2 (5%)	√	Q2	√	Q1
*International Journal of Human-Computer Interaction*	2 (5%)	√	Q1	√	Q1
*Information Systems Management*	2 (5%)	√	Q1	√	Q1
*Sustainability*	2 (5%)	√	Q2	√	Q1
*Architectural Engineering and Design Management*	1 (2.5%)	√	Q2	√	Q1
*Asian Journal of Business and Accounting*	1 (2.5%)	√	-	√	Q3
*Complexity*	1 (2.5%)	√	Q2	√	Q2
*Health Informatics Journal*	1 (2.5%)	√	Q3	√	Q2
*International Journal of Information Management*	1 (2.5%)	√	Q1	√	Q1
*Interdisciplinary Journal of Information, Knowledge, and Management*	1 (2.5%)	-	-	√	Q2
*Internet Research*	1 (2.5%)	√	Q1	√	Q1
*International Journal of Knowledge Management Education*	1 (2.5%)	√	Q1	√	Q1
*International Journal of Knowledge Management Studies*	1 (2.5%)	√	-	√	Q3
*International Journal of Information and Communication Technology Education*	1 (2.5%)	√	-	√	Q2
*Information Processing and Management*	1 (2.5%)	√	Q1	√	Q1
*Journal of Computer Information Systems*	1 (2.5%)	√	Q3	√	Q1
*Journal of Global Entrepreneurship Research*	1 (2.5%)	√	-	-	-
*Journal of Research in Interactive Marketing*	1 (2.5%)	√	Q1	√	Q1
*Journal of the Association for Information Science and Technology*	1 (2.5%)	√	Q2	√	Q1
*Journal of Modern Project Management*	1 (2.5%)	-	-	√	Q3
*Kybernetes*	1 (2.5%)	√	Q3	√	Q2
*Library Hi Tech*	1 (2.5%)	√	Q2	√	Q2
*Management Decision*	1 (2.5%)	√	Q3	√	Q1
*PLoS ONE*	1 (2.5%)	√	Q2	√	Q1
*Technological Forecasting and Social Change*	1 (2.5%)	√	Q1	√	Q1
*Tourism Management*	1 (2.5%)	√	Q1	√	Q1
*Universal Access in the Information Society*	1 (2.5%)	√	Q3	√	Q2
*VINE Journal of Information and Knowledge Management Systems*	1 (2.5%)	-	-	√	Q1

**Table 2:** The conceptual/theoretical frameworks utilized by the reviewed articles.

Conceptual/Theoretical Framework	Study ID	No.
Theory of Planned Behavior ^1^	[9,61,65,66,72,86]	6
Technology Acceptance Model ^1^	[9,45,69,70,72]	5
Social Cognitive Theory ^1^	[41,67,75,77]	4
Intrinsic and Extrinsic Motives ^1^	[46,61,74,77]	4
Unified Theory of Acceptance and Use of Technology ^1^	[63,81,83,84]	4
Theory of Reasoned Action ^1^	[59,65,90]	3
Task-Technology Fit Theory ^1^	[88]	1
Expectation Confirmation Theory ^1^	[65]	1
Information Systems Continuous Use Model ^1^	[73]	1
Valance, Instrumentality and Expectancy ^1^	[60]	1
Expectation Disconfirmation Theory ^1^	[88]	1
Social Exchange Theory ^2^	[59,62,74,87]	4
Commitment Trust Theory ^2^	[62,73]	2
Commitment Model ^2^	[87]	1
Social Identity Theory ^2^	[66]	1
Social Influence Theory ^2^	[76]	1
Stimulus–Organism–Response Framework ^2^	[26]	1
Utility Interdependence ^2^	[58]	1
*Not utilizing a conceptual framework*	[36,44,64,68,71,78,79,80,82,85]	10

^1^ Personal Behavior Theories. ^2^ Social Behavior Theories.

**Table 3:** Summary of factors influencing intentions to share knowledge on social media.

Study ID	Key Issues	Findings
[9]	Knowledge-sharing intention	Attitude, subjective norm, perceived behavioral control, and interpersonal trust
[23]	Knowledge-sharing intention	Social capital and social identity
[36]	Knowledge-sharing behavior	Belief in integrity and perceived ease of use
[41]	Knowledge-sharing intention	Enjoyment, fairness, identification, and reciprocity
[44]	Knowledge-sharing intention	Subjective well-being
[45]	Advertisement-sharing intention	Advertisement content likeability
[46]	Music-sharing intention	Perceived enjoyment, self-presentation, relationship development, social identity, social presence, face risk, and perceived total benefit
[58]	Knowledge-sharing behavior	Community’s need for knowledge, foregone benefit of free riding, knowledge codification effort, and level of knowledge
[59]	Knowledge-sharing intention	Attitude
[60]	Knowledge-sharing behavior	Importance of knowledge exchange, perceived usefulness of social media, and experience using social media
[61]	Knowledge-sharing intention	Attitudes, subjective norms, and perceived behavior control
[62]	Knowledge-sharing behavior	Relationship commitment
[63]	Knowledge-sharing behavior	Performance expectancy, effort expectancy, and social influence
[64]	Knowledge-sharing behavior	Satisfaction
[65]	Knowledge-sharing behavior	Satisfaction
[66]	Knowledge-sharing behavior	Personal online identity, web-specific self-efficacy, and knowledge-creating self-efficacy
[67]	Knowledge-sharing intention	Outcome expectations and community climate
[68]	Knowledge-sharing behavior	Commitment, trust, and knowledge-sharing self-efficacy
[69]	Knowledge-sharing behavior	Perceived usefulness, managerial support, and altruism
[70]	Knowledge-sharing intention	Trust
[71]	Knowledge-sharing behavior	Leader membership exchange, perceived organisation support, Homan Proposition (Concern for others), and institutional factors
[72]	Knowledge-sharing intention	Attitude and perceived behavioral control
[73]	Knowledge-sharing intention	Affective commitment, satisfaction, and trust
[74]	Knowledge-sharing behavior	Attitude
[75]	Knowledge-sharing behavior	Social capital
[76]	Knowledge-sharing intention	Subjective norm, group norm, and social identity
[77]	Knowledge-sharing intention	Intrinsic motivation and extrinsic motivation
[78]	Knowledge-sharing behavior	Community trust and community identification
[79]	Knowledge-sharing intention	Virtual network connectivity
[80]	Knowledge-sharing behavior	Community attachment
[81]	Knowledge-sharing behavior	Performance expectancy and hedonic motivation
[82]	Knowledge-sharing behavior	Trust, reciprocity, altruism, and reputation
[83]	Knowledge-sharing behavior	Performance expectancy and self-efficacy
[84]	Knowledge-sharing intention	Performance expectancy, effort expectancy, social influence, and attitude
[85]	Knowledge-sharing intention	Source credibility, content credibility, and institution-based trust
[86]	Knowledge-sharing behavior	Material reward and self-efficacy
[87]	Knowledge-sharing behavior	Affective commitment and normative commitment
[88]	Knowledge-sharing behavior	Knowledge-related task–technology fit, satisfaction, and knowledge self-efficacy
[89]	Knowledge-sharing intention	Perceived usefulness and perceived ease of use
[90]	Knowledge-sharing behavior	Attitude, subjective norms, and enjoyment in helping others

The above factors positively impact the intentions to share knowledge on social media.

**Table 4:** A summary of psychological factors.

Psychological Factors	Study ID	No.
Trust	[9,68,70,73,78,82,85]	7
Attitude	[9,59,61,72,74,84,90]	7
Self-efficacy	[66,68,83,86,88]	5
Satisfaction	[64,65,73,88]	4
Commitment	[62,68,73,87]	4
Perceived behavioral control	[9,61,72]	3
Altruism ^1^	[69,82,90]	3
Perceived enjoyment ^1^	[41,46,81]	3
Identification ^1^	[41,66]	2
Perceived benefit ^1^	[46,58]	2
Intrinsic motivation ^1^	[77]	1
Extrinsic motivation ^1^	[77]	1
Reciprocity ^1^	[82]	1
Belief in integrity ^1^	[37]	1
Self-presentation ^1^	[46]	1
Material reward ^1^	[86]	1
Concern for others ^1^	[71]	1
Relationship development ^1^	[46]	1
Reputation ^1^	[82]	1
Importance of knowledge exchange ^2^	[60]	1
Level of knowledge ^2^	[58]	1
Source credibility ^2^	[85]	1
Content credibility ^2^	[85]	1
Knowledge codification effort ^2^	[58]	1
Advertisement content likeability ^2^	[45]	1

^1^ Motivation. ^2^ Knowledge.

**Table 5:** A summary of technology factors.

Technology Factors	Study ID	No.
Performance expectancy	[63,67,81,83,84]	5
Perceived usefulness	[60,69,89]	3
Effort expectancy	[63,84]	2
Perceived ease of use	[36,89]	2
Knowledge-related task–technology fit	[88]	1
Virtual network connectivity	[79]	1
Face risk	[46]	1
Experience using social media	[60]	1

**Table 6:** A summary of environmental factors.

Environmental Factors	Study ID	No.
Group norm	[76]	1
Community climate	[67]	1
Community’s need for knowledge	[58]	1
Community identification	[78]	1
Community attachment	[80]	1
Institutional factors	[71]	1
Managerial support	[69]	1
Perceived organization support	[71]	1
Leader membership exchange	[71]	1
Fairness	[41]	1

**Table 7:** A summary of social factors.

Social Factors	Study ID	No.
Subjective norm	[9,61,76,90]	4
Social influence	[46,63,84]	3
Social identity	[23,46,76]	3
Social capital	[23,75]	2

**Figure A1:**
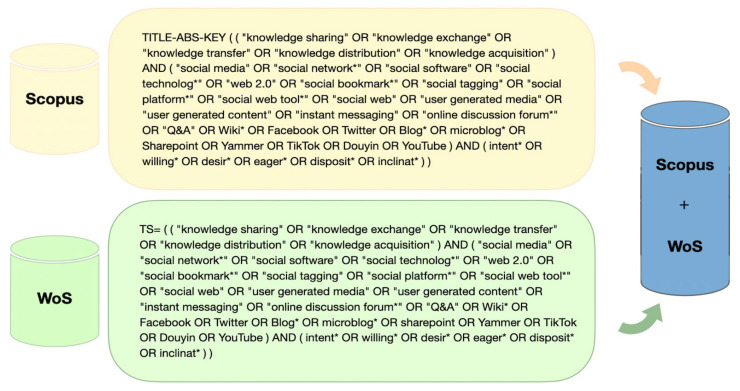
An illustration of a full-search approach. Notes: Wos, Web of Science; TITLE-ABS-KEY, Title, Abstract, Keywords; TS, Topic—searches Title, Abstract, Author Keywords, Keywords Plus. The asterisk (*) at the end of a search term accepts any variant of a base term. Quotation marks are used to search the exact word order (or phrase). Boolean operators “OR” and “AND” ensure that at least one term appears between the parentheses.

**Figure A2:**
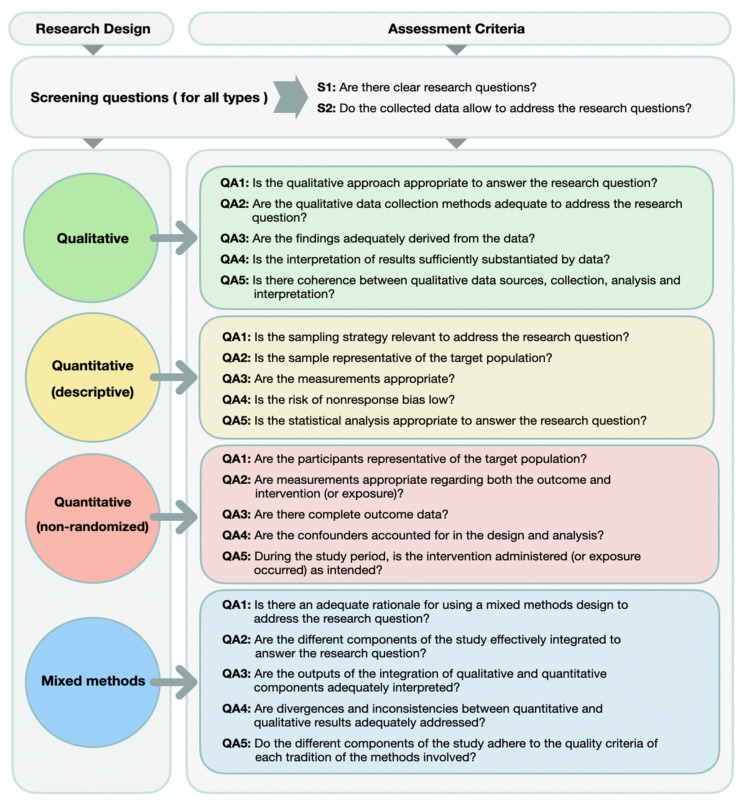
Criteria for selected articles.

## Data Availability

The data in this study can be obtained through the screening process in Section 2.3 of this article.

## Appendix A. Question Search Strategy

### Appendix A.1. Stage 1: Identification

We systematically reviewed the literature using the Scopus and Web of Science databases. Scopus includes a broader range of conference papers and journal coverage [95], while Web of Science encompasses high-impact journals and disciplines [96]. By utilizing both Scopus and Web of Science databases, we could more comprehensively, accurately, and systematically identify and collect studies for the literature review, thereby enhancing the quality and credibility of the review [97]. Then, we defined the terms used in the search strings according to the research questions to obtain the most relevant results from the preliminary literature search. We searched the Scopus and Web of Science databases using “TITLE-ABS-KEY” and “TS” as field tags, respectively. The query used in both cases is shown in Figure A1. After an exhaustive search, we identified 1160 potential articles in the selected databases.

### Appendix A.2. Stage 2: Screening

The screening process involved evaluating articles to determine whether they met the criteria and were of sufficient quality for inclusion in the study (see Table A1). An initial screening was performed through the database, followed by a more comprehensive manual screening of the articles. We focused exclusively on articles published between 2015 and 2023, consistent with the concept of maturity of the research field, emphasized by Kraus et al. [98] In other words, selecting literature in this range ensures that the latest and most relevant research is covered. We also limited our investigation to articles published in English. Additionally, we excluded conference papers, conference abstracts, book chapters, book reviews, notes, news, and letters, including only research journal articles containing primary data. We also manually performed a comprehensive literature search for studies related to KSIoSM based on our search criteria. During this assessment phase, 640 out of 1160 articles were disqualified because they did not meet the inclusion criteria. Consequently, 520 items remained for examination in the subsequent step.

**Table A1:** Inclusion and exclusion criteria.

Criterion	Inclusion	Exclusion
Timeline	2015–2023	2014 and earlier
Document type	Articles (with empirical data)	Books, article summaries, prefaces, interviews, news, reviews, etc.
Full-text acquisition	Full text available	Full text not available
Language	English	Non-English
Research objective	Directly answered the research question	Unrelated research purposes

### Appendix A.3. Stage 3: Eligibility

The process of excluding articles was conducted in three phases. During the initial phase, 137 duplicates were identified by importing 520 relevant articles from the two databases into the Mendeley software 1.19.8, with the remaining 383 articles proceeding to the next phase. For phase two, a thorough examination of article titles, abstracts, and contents was conducted, resulting in 276 articles being excluded as irrelevant to KSIoSM. During the third phase, the complete papers were acquired and meticulously examined. Fifty papers were rejected during this phase for not focusing on elements influencing individual KSIoSM. Another 11 articles were excluded because we could not access the complete text, and six were excluded because they were repetitive. Consequently, 40 articles were retained. Relevant data were extracted from the articles and entered into an Excel spreadsheet, encompassing the author’s name, publication year, title, journal name, social media platforms, theoretical frameworks employed, study location, and research outcomes.

## Appendix B. Quality Appraisal

The assessment criteria we relied upon to develop our quantitative research are outlined in Figure A2. The criteria proposed by Hong et al. [51] include twenty-five specific assessment items (five for each methodological domain), along with two screening questions common to all study types (S1 and S2 in Figure A2). Additionally, if a negative answer is given to one screening question or uncertain answers are given to both, the study should be considered ineligible or not easily assessed by this tool [51]. Given our mixed-methods research design, the MMAT has helped to ensure methodological and analytical rigor, as well as guiding us through several critical domains, including the validity of different research designs for addressing research questions, the fundamentals of using mixed methods to investigate research questions, the seamless combination of qualitative and quantitative components, and the importance of the study and its ability to deal with differences between the various research designs. The two researchers comprehensively examined each article, paying particular attention to the methodology and analysis sections. Using the MMAT tool, we evaluated the appropriateness of the sampling techniques and analysis methods (see Table A2), including random sampling and inferential analysis [51]. We used five-point assessment criteria to analyze the research question in each article, and papers were deemed eligible for inclusion if they satisfied a minimum of three criteria. Any disagreement between assessments was resolved through researcher deliberation. The final sample represents a consensus between the two researchers on articles that met the quality standards of this review for research methods and analysis. The 19 papers selected for this systematic review satisfied all the stated standards, whereas 15 articles fulfilled a minimum of four criteria. Furthermore, six articles met a minimum of three requirements, as illustrated in Table A2.

**Table A2:** Results of the quality assessment.

Study ID	RD	QA1	QA2	QA3	QA4	QA5	NF	IR
[9]	QD	√	√	√	√	√	5/5	√
[23]	QD	√	√	√	√	√	5/5	√
[36]	QD	√	√	√	√	√	5/5	√
[41]	QD	√	√	√	√	√	5/5	√
[44]	QD	√	X	√	√	√	4/5	√
[45]	QD	√	X	√	√	√	4/5	√
[46]	QD	√	X	√	C	√	3/5	√
[58]	QD	√	√	√	√	√	5/5	√
[59]	QD	√	X	√	X	√	3/5	√
[60]	QD	√	X	√	C	√	3/5	√
[61]	QD	√	√	√	√	√	5/5	√
[62]	QD	√	X	√	√	√	4/5	√
[63]	QD	√	√	√	√	√	5/5	√
[64]	QD	√	√	√	√	√	5/5	√
[65]	QD	√	√	√	√	√	5/5	√
[66]	QD	√	√	√	√	√	5/5	√
[67]	QD	√	C	√	√	√	4/5	√
[68]	QD	√	√	√	√	√	5/5	√
[69]	QD	√	X	√	√	√	4/5	√
[70]	QD	√	X	√	√	√	4/5	√
[71]	MM	X	√	√	C	√	3/5	√
[72]	QD	√	√	√	√	√	5/5	√
[73]	QD	√	√	√	√	√	5/5	√
[74]	QD	√	C	√	√	√	4/5	√
[75]	QD	√	X	√	√	√	4/5	√
[76]	QD	√	X	√	X	√	3/5	√
[77]	QD	√	√	√	√	√	5/5	√
[78]	QD	√	√	√	√	√	5/5	√
[79]	QD	√	√	√	√	√	5/5	√
[80]	QD	√	√	√	√	√	5/5	√
[81]	QD	√	√	√	√	√	5/5	√
[82]	QD	√	C	√	√	√	4/5	√
[83]	QD	√	√	√	X	√	4/5	√
[84]	QD	√	X	√	X	√	3/5	√
[85]	QD	√	C	√	√	√	4/5	√
[86]	QD	√	X	√	√	√	4/5	√
[87]	QD	√	X	√	√	√	4/5	√
[88]	QD	√	√	√	√	√	5/5	√
[89]	MM	√	√	√	C	√	4/5	√
[90]	QD	√	X	√	√	√	4/5	√

Abbreviations: RD, research design; QA, quality assessment; QD, quantitative descriptive; MM, mixed-methods; C, cannot tell; NF, number of criteria fulfilled; IR, inclusion in the review.
